# FoxO3a: capture the bond between magnesium and diabetic kidney disease

**DOI:** 10.3389/fendo.2025.1603150

**Published:** 2025-08-13

**Authors:** Taoran Chen, Miao Sun, Qi Zhou, Jiancheng Xu

**Affiliations:** ^1^ Department of Laboratory Medicine, First Hospital of Jilin University, Changchun, China; ^2^ Department of Pediatrics, First Hospital of Jilin University, Changchun, China

**Keywords:** FoxO3a, magnesium, oxidative stress, ferroptosis, diabetic kidney disease

## Abstract

Hyperglycemia in Diabetic Kidney Disease (DKD) induces excessive accumulation of reactive oxygen species (ROS) through various pathways, leading to oxidative stress, ferroptosis, and mitochondrial dysfunction, which collectively contribute to kidney damage. Currently, the treatment of DKD remains a significant challenge. Magnesium, an essential mineral, has emerged as a promising therapeutic agent for DKD due to its anti-inflammatory and antioxidant properties. Magnesium has been shown to alleviate renal fibrosis, maintain tubular integrity and function, improve endothelial cell function, and regulate renal hemodynamics. As a cofactor of antioxidant enzymes, Magnesium directly scavenges ROS and enhances the expression of antioxidant proteins. This review explores the relationship between Magnesium and DKD, examining how Magnesium mitigates oxidative stress through the PI3K/AKT/FoxO3a pathway, inhibits ferroptosis in renal tubular epithelial cells via the AMPK/FoxO3a/Nrf2 pathway, and reduces autophagy and apoptosis, thereby delaying DKD progression. The review further discusses how Magnesium regulates the pivotal FoxO3a protein, a transcription factor with antioxidant properties, leading to the prevention of DKD, and proposes Magnesium supplementation as a potential clinical strategy for alleviating DKD, offering a new therapeutic approach for its treatment.

## 1 Introduction

Diabetic Kidney Disease (DKD) is a progressive kidney disease and a common complication of diabetes mellitus. Persistent proteinuria (>300 mg/day or >200 μg/minute) on at least two occasions over a period of three to six months after diabetes diagnosis indicates the onset of DKD, which is accompanied by a gradual decline in glomerular filtration rate. DKD is considered the leading cause of chronic kidney disease and end-stage renal failure (1).

The progression of DKD is closely linked to oxidative stress and cell death induced by hyperglycemia (2). Oxidative stress, in turn, promotes an inflammatory response, creating an oxidative stress-inflammatory cycle that damages renal podocytes, endothelial cells, and tubular epithelial cells (3). The pathogenesis of DKD is related to metabolic irregularities, oxidative stress, and iron-mediated damage, each contributing to the progression of DKD either independently or synergistically (4). Therefore, a comprehensive investigation of the underlying mechanisms of DKD is crucial for identifying potential therapeutic targets and developing more effective treatment strategies and pharmacological interventions for DKD.

Magnesium, an essential mineral, plays a key role in numerous physiological processes and has been shown to have protective effects against DKD (5). Magnesium supplementation has been associated with improved prognosis in both animal and clinical studies focused on diabetes and its complications (6, 7). Furthermore, Magnesium, as an important regulator of antioxidants, may influence ferroptosis, warranting further investigation. Therefore, understanding the molecular mechanisms of Magnesium in DKD and exploring its therapeutic significance are essential for developing novel strategies to treat DKD and related disorders.

Members of the FoxO family, FoxO1, FoxO3, FoxO4, and FoxO6, are expressed in various body tissues and involved in the regulation of various cellular functions. FOXO proteins are highly involved in diabetic complications as they are responsible for regulation of oxidative stress, apoptosis, autophagy, inflammation, etc., which are also the major underlying causes in the development of diabetic complications (8, 9).

Therefore, this review elucidates the relationship between Magnesium and DKD, as well as the interactions between the FoxO family and Magnesium in the pathogenesis of DKD. This review discusses the alleviation by Magnesium of oxidative stress via the FoxO3a pathway, the inhibition of ferroptosis in renal tubular epithelial cells through the AMPK/FoxO3a/Nrf2 pathway. This review also further details the effect of Magnesium on the pathogenesis of DN via the FoxO3a protein, a transcription factor with antioxidant properties. At the end of the review, we also discuss the potential supplementation of Magnesium as a clinical approach to alleviate DN.

This review was conducted in accordance with the PRISMA (Preferred Reporting Items for Systematic Reviews and Meta-Analyses) guidelines. A comprehensive literature search was performed using PubMed, Web of Science, and Scopus, covering publications from January 2012 to December 2024.

The search strategy included a combination of the following keywords and Medical Subject Headings (MeSH), using Boolean operators: “Magnesium” AND “diabetic kidney disease” AND “FoxO3a” AND (“oxidative stress” OR “ferroptosis”) AND (“PI3K/Akt” OR “AMPK” OR “Nrf2”) AND “Magnesium supplementation”.

The inclusion criteria were: Peer-reviewed original research articles and review papers; studies published in English; articles focusing on the role of Magnesium, FoxO3a, oxidative stress, and ferroptosis in diabetic nephropathy.

The exclusion criteria included: Conference abstracts, editorials, case reports, and commentaries; studies irrelevant to DN or lacking mechanistic discussion related to Magnesium or FoxO3a.

**Figure 1:**
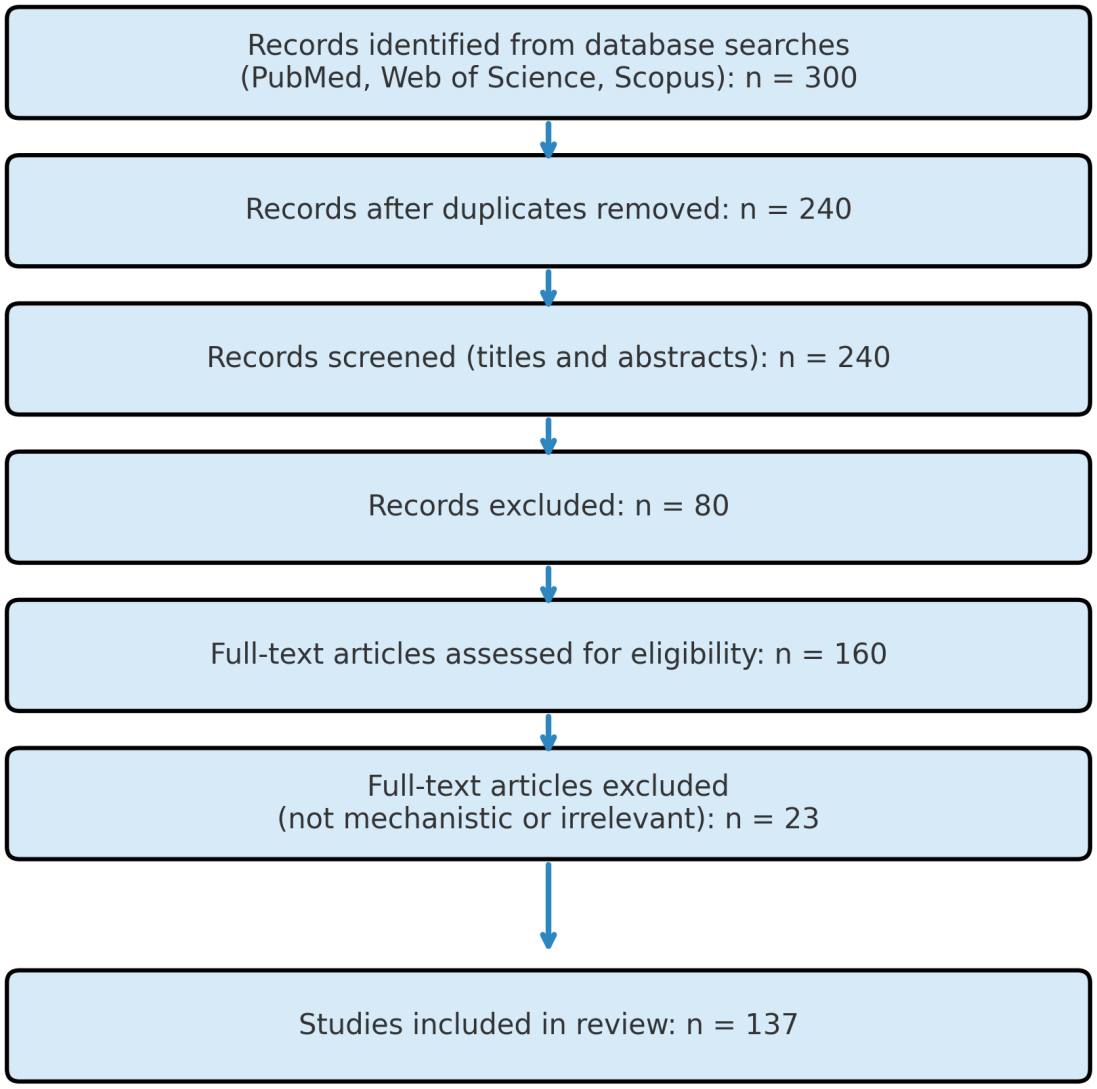
PRISMA flow diagram illustrating the study-selection process. Records (n = 300) were retrieved from PubMed, Web of Science, and Scopus using the search string “Magnesium” AND “diabetic nephropathy” AND “FoxO3a” AND (“oxidative stress” OR “ferroptosis”) AND (“PI3K/Akt” OR “AMPK” OR “Nrf2”) AND “magnesium supplementation” (2012 – 2024). After removing duplicates (n = 60), 240 records were screened by title and abstract; 80 were excluded as irrelevant. The full texts of the remaining 160 articles were evaluated in detail, and 23 were excluded for lacking mechanistic data or relevance to magnesium/FoxO3a in diabetic nephropathy. A total of 137 studies met all inclusion criteria and were incorporated into the present review.

A total of 300 articles were identified. After removing 60 duplicates, 240 records remained. Following title and abstract screening, 80 articles were excluded. After full-text assessment, an additional 23 articles were excluded due to irrelevance or insufficient mechanistic detail. Finally, 137 studies were included in this review. The selection process is outlined in **Figure 1** (PRISMA Flow Diagram).

## 2 Magnesium and DKD

Magnesium ions (Magnesium) are important regulators of enzymes involved in glycolysis, assisting in the regulation of adenine nucleotides. Additionally, Magnesium plays a role in regulating insulin secretion. A study has shown that average Magnesium levels in patients with diabetes are significantly lower compared to non-diabetic individuals (10). This suggests a close relationship between Magnesium and DKD.

A decade ago, study have shown that patients with type 2 diabetes mellitus exhibit serum Magnesium deficiency and elevated urinary Magnesium excretion, and the deficient Magnesium metabolism also related to the disease progression and development of complications (11). The study also demonstrated that serum Magnesium levels are also significantly reduced in a mouse model of type 1 diabetes mellitus (12). Additionally, a study with animals has demonstrated that Magnesium deficiency induces an inflammatory response and overactivates leukocytes and macrophages, leading to the overproduction of inflammatory cytokines, acute-phase proteins, and ROS, which increase the incidence and severity of DKD (13). These findings confirm reduced serum Magnesium levels in diabetes; however, whether Magnesium deficiency exacerbates diabetes progression and promotes DKD remains to be fully explored. The study has shown that serum Magnesium concentration in DKD patients is negatively correlated with serum creatinine levels, while urinary Magnesium concentration is positively correlated with estimated glomerular filtration rate (eGFR) (11). Moreover, serum Magnesium levels decrease with renal impairment in DKD patients (5). There is also growing evidence that Magnesium deficiency affects the tricarboxylic acid (TCA) cycle, increases the risk of hyperinsulinemia, and leads to insulin resistance (14), thereby exacerbating DKD progression. Furthermore, Magnesium deficiency affects lipid metabolism and the antioxidant system, contributing to the development of other metabolic disorders. Previous studies indicate that low serum Magnesium is significantly associated with DKD and can serve as a risk marker for predicting DKD development (14, 15). In summary, a bidirectional relationship between Magnesium deficiency and DKD has been demonstrated. Magnesium deficiency leads to impaired glucose tolerance and insulin resistance, contributing to DKD progression and worsening its prognosis. Furthermore, DKD patients are significantly associated with hypomagnesemia, and Magnesium can serve as a predictor for DKD development (15, 16). Current clinical trials indicate that Magnesium has a nephroprotective role in DKD (17, 18); however, further research is needed to elucidate the specific mechanisms through which Magnesium regulates DKD.

## 3 Mechanisms of magnesium regulating cell physiological process to improve DKD

The aforementioned studies confirm that Magnesium deficiency causes oxidative stress and exacerbates diabetes progression and DKD-related kidney damage. Magnesium may alleviate DKD by regulating physiological and pathological processes, including oxidative stress, ferroptosis, and mitochondrial apoptosis (14, 18, 19).

### 3.1 Regulating oxidative stress

Oxidative stress refers to the excessive accumulation of ROS resulting from an imbalance between oxidative and antioxidant systems, ultimately leading to cellular damage. One typical consequence of excessive ROS accumulation is lipid peroxidation, which damages cell membranes and impairs renal cell function (20), thereby promoting DKD progression. In DKD, persistent hyperglycemia is the primary cause of oxidative stress, leading to an excessive increase in ROS that overwhelms the body’s antioxidant capacity. This induces DNA and protein damage, alters renal tubular structure and function (21), and ultimately exacerbates renal and systemic damage (22).

In summary, oxidative stress accelerates glomerular injury, tubular atrophy, and interstitial fibrosis, ultimately resulting in decreased renal function and renal failure. Therefore, alleviating oxidative stress is crucial for treating DKD. Clinical treatments targeting oxidative stress are now emerging. Magnesium as a treatment was reported to considerably reduce insulin resistance and blood glucose levels, as well as attenuate renal hypertrophy and ameliorate associated inflammation in rats with DKD (23, 24.

Magnesium is an essential mineral with multiple physiological functions and plays a potential role in mitigating oxidative stress, primarily by regulating various cellular and enzymatic processes in the antioxidant defense mechanism. One mechanism through which Magnesium alleviates oxidative stress is by leveraging its antioxidant properties. Magnesium is involved in numerous enzymatic reactions *in vivo* and exhibits antioxidant, anti-inflammatory, and anti-apoptotic effects (25). Magnesium modulates the expression and activity of several proteins involved in oxidative stress regulation to confer resistance against oxidative damage. Magnesium acts as a cofactor for antioxidant enzymes such as superoxide dismutase (SOD), which converts superoxide radicals into less reactive substances; glutathione peroxidase (GPX), which detoxifies lipid peroxides with the help of glutathione (GSH). By enhancing these enzyme activities, Magnesium helps prevent oxidative damage. In addition, Magnesium possesses anti-inflammatory properties. Inflammation induces oxidative stress, which, in turn, exacerbates the inflammatory response, creating a cycle of mutual reinforcement. Studies have shown that Magnesium inhibits the production of pro-inflammatory cytokines such as interleukin-6 (IL-6) and tumor necrosis factor-alpha (TNF-α), as well as the activation of NF-κB, a key transcription factor in the inflammatory response (6, 26). By reducing inflammation, Magnesium indirectly reduces ROS production and oxidative stress damage. Furthermore, ferroptosis, a form of regulated cell death closely linked to oxidative stress, also contributes to the onset and progression of DKD.

### 3.2 Regulating ferroptosis

Ferroptosis is a recently identified non-apoptotic form of cell death, characterized by lipid peroxidation due to intracellular iron accumulation and the regulation of multiple genes and proteins (27).

At least four defense mechanisms against cellular ferroptosis have been identified (28, 29), including the cytoplasmic and GSH-GPX4 system, the cytoplasmic ferroptosis suppressor protein 1 (FSP1)-coenzyme Q10 (CoQ10) system, the mitochondrial dihydroorotate dehydrogenase (DHODH)-CoQ system, and the guanosine triphosphate cyclohydrolase 1 (GCH1)-tetrahydrobiopterin (BH4) system. (1) The GSH-GPX4 system (30) is the primary defense mechanism against ferroptosis. It imports extracellular cystine via the cystine-glutamate antiporter (composed of SLC7A11 and SLC3A2), which is then reduced to cysteine intracellularly for the synthesis of the major antioxidant, glutathione (GSH). GPX4, a key regulator of ferroptosis, uses GSH as its main cofactor to neutralize lipid peroxides, thereby inhibiting ferroptosis. (2) The FSP1-CoQ10 system (31) inhibits ferroptosis independently of the GSH-GPX4 pathway. FSP1 functions as an NAD(P)H-dependent oxidoreductase that reduces CoQ10 to ubiquinol (CoQH2), which prevents lipid peroxidation and ferroptosis by neutralizing lipid peroxyl radicals. (3) In the DHODH-CoQ system (32), DHODH reduces CoQ to CoQH2 in the inner mitochondrial membrane. When GPX4 is inactivated, DHODH-mediated CoQH2 production is enhanced, neutralizing lipid peroxidation and preventing ferroptosis. (4) In the GCH1-BH4 system (33), BH4 acts as an antioxidant capable of capturing lipid peroxyl radicals, and GCH1 mediates the rate-limiting step of BH4 synthesis. Additionally, GCH1 promotes CoQH2 production to defend against ferroptosis. The DHODH-CoQ and GCH1-BH4 systems function in mitochondria, with the DHODH-CoQ mechanism being fundamentally similar to that of FSP1-CoQ10, both reducing CoQ to CoQH2.

DKD is one of the most severe complications of diabetes. Persistent hyperglycemia stimulates the renal vasculature, altering renal structure and function, and leading to metabolic dysfunction (34). Following metabolic dysfunction, interactions among oxidative stress, hemodynamic changes, and immune dysregulation lead to renal cell apoptosis, autophagy, pyroptosis, and ferroptosis. Ferroptosis damages β-cells and impairs insulin secretion, and also affects key renal cells, including tubular epithelial cells (TECs), podocytes, glomerular endothelial cells (GECs), and glomerular mesangial cells, thereby promoting DKD progression (35–38). In DKD, hyperglycemia leads to an overload of redox-active iron (Fe^2+^) and an overaccumulation of ROS, creating favorable conditions for ferroptosis. Additionally, studies have shown that GPX4 expression is reduced to varying degrees in both cells and kidneys in DKD models, weakening cellular resistance to oxidative stress and promoting lipid peroxidation, ultimately leading to ferroptosis (39).

Recent studies using cellular and animal models have confirmed that inhibiting ferroptosis in renal cells slows the progression of DKD. Mesangial cell damage is a fundamental pathological feature of DKD (40). The study has shown that inhibiting ferroptosis in renal mesangial cells can alleviate DKD (38). Secondly, ferroptosis is also associated with podocyte damage in DKD patients. Podocytes are a crucial component of the glomerular filtration barrier (GFB) and have been found to regulate the GFB through endocytosis. Podocyte injury is considered a primary mechanism underlying GFB damage. The absence or inhibition of key ferroptosis-preventing proteins, such as GPX4 and SLC7A11, can induce ferroptosis. *In vitro* studies have shown that upregulating the expression of these proteins can prevent podocyte injury in DKD (41). Additionally, studies have demonstrated that inhibiting ferroptosis in renal tubular epithelial cells reduces diabetes-induced tubular injury, thereby treating DKD (39, 42). As a newly discovered form of cell death, ferroptosis plays a crucial role in DKD and offers a novel therapeutic approach for DKD patients.

In the process of ferroptosis, Magnesium acts as a crucial cofactor for enzymes involved in glutathione (GSH) production—such as glutamate-cysteine ligase catalytic subunit (GCLC), glutamate-cysteine ligase modifier subunit (GCLM), glutathione disulfide reductase (GSR), and GSH synthase (GSS)—and also activates transcription factors to regulate GPX gene and protein expression (43). Additionally, Magnesium upregulates the expression of Nrf2, a key transcriptional activator of antioxidant responses. This enhances the ferroptosis defense system by increasing the synthesis of endogenous antioxidants, mitigates cellular damage and inflammatory responses in the hyperglycemic state of DKD patients, and delays DKD progression.

### 3.3 Regulating mitochondrial apoptosis

Apoptosis plays a critical role in removing abnormal cells from the body and can occur through two primary pathways: the extrinsic pathway, mediated by apoptosis receptors, and the intrinsic pathway, mediated by mitochondria and the endoplasmic reticulum. Renal proximal tubular cells are rich in mitochondria (44). Therefore, the mitochondria-mediated intrinsic apoptotic pathway in the renal tubular system plays a key role in DKD.

The mitochondria-mediated intrinsic apoptosis pathway begins with the accumulation of the pro-apoptotic protein BAX in the mitochondrial membrane, followed by the release of cytochrome c into the cytoplasm and activation of the caspase cascade, leading to mitochondrial dysfunction, decreased mitochondrial redox levels, and increased intracellular ROS, ultimately resulting in DNA damage and cell death (18, 45). Mitochondria play a critical role in the intrinsic apoptosis pathway (46, 47). They are targets of BCL-2 family proteins, which are key regulators of the initiation of intrinsic apoptosis (47, 48), and serve as resident targets for certain intracellular apoptotic proteins (49).

Studies have shown that mitochondria serve as crucial intracellular Magnesium stores, and Magnesium plays a role in regulating mitochondrial function (50, 51). At the cellular level, Magnesium enhances mitochondrial function by increasing ATP production, decreasing mitochondrial ROS and intracellular calcium (Ca^2+^) overload, repolarizing the mitochondrial membrane, and reducing ROS production. Previous studies have demonstrated that Magnesium is crucial for most glycolytic enzymes due to its ability to form Magnesium -ATP2 complexes (52). Magnesium has been shown to enhance the activities of three mitochondrial dehydrogenases involved in energy metabolism, thereby regulating intracellular oxidative phosphorylation. Additionally, Magnesium acts as an activator of ATP synthesis through mitochondrial F0/F1-ATPase (53).

Beyond the cellular level, Magnesium can alleviate mitochondrial apoptosis by stimulating mitochondrial enzymes, modulating mitochondrial Ca^2+^ transport, and reducing pro-inflammatory cytokine levels in renal cells. Magnesium also influences mitochondrial function and metabolic state by stimulating mitochondrial enzymes, which subsequently affect Magnesium concentrations in the matrix and cytoplasm (54, 55). A bidirectional interaction appears to exist between Magnesium and mitochondrial energy metabolism. The influence of Magnesium on energy metabolism also impacts mitochondrial Ca^2+^ transport. Mitochondria play a critical role in intracellular Ca^2+^ homeostasis and signal transduction (56). Mitochondria actively accumulate and release intracellular Ca^2+^ through various Ca^2+^ channels on the mitochondrial membrane. Magnesium can enhance energy metabolism and mitigate apoptosis by inhibiting mitochondrial Ca^2+^ channels. The role of Magnesium in apoptosis is often overlooked in comparison to Ca^2+^. By inhibiting Ca^2+^ channels, Magnesium further restricts Ca^2+^ entry into cells, reduces levels of apoptosis-related proteins (e.g., caspase-3 and BAX), and increases the expression of anti-apoptotic proteins such as BCL-2 (57). A related animal study has demonstrated that alleviating podocyte apoptosis in rat glomeruli restores renal function, mitigates glomerular injury, and reduces symptoms such as proteinuria (58). Additionally, elevated pro-inflammatory cytokine levels can activate caspase-3 and oxidative stress, while NF-κB regulates the expression of downstream BCL-2 genes and apoptosis (53). In fetal rats, Magnesium significantly reduces intracellular levels of inflammatory factors such as caspase-3, NF-κB, interleukin-6 (IL-6), and tumor necrosis factor-alpha (TNF-α), and inhibits NF-κB acetylation, thereby reducing apoptosis (59, 60). In a rat model of renal reperfusion injury, studies confirmed that Magnesium inhibited caspase-3 activity and apoptosis (61).

**Figure 2:**
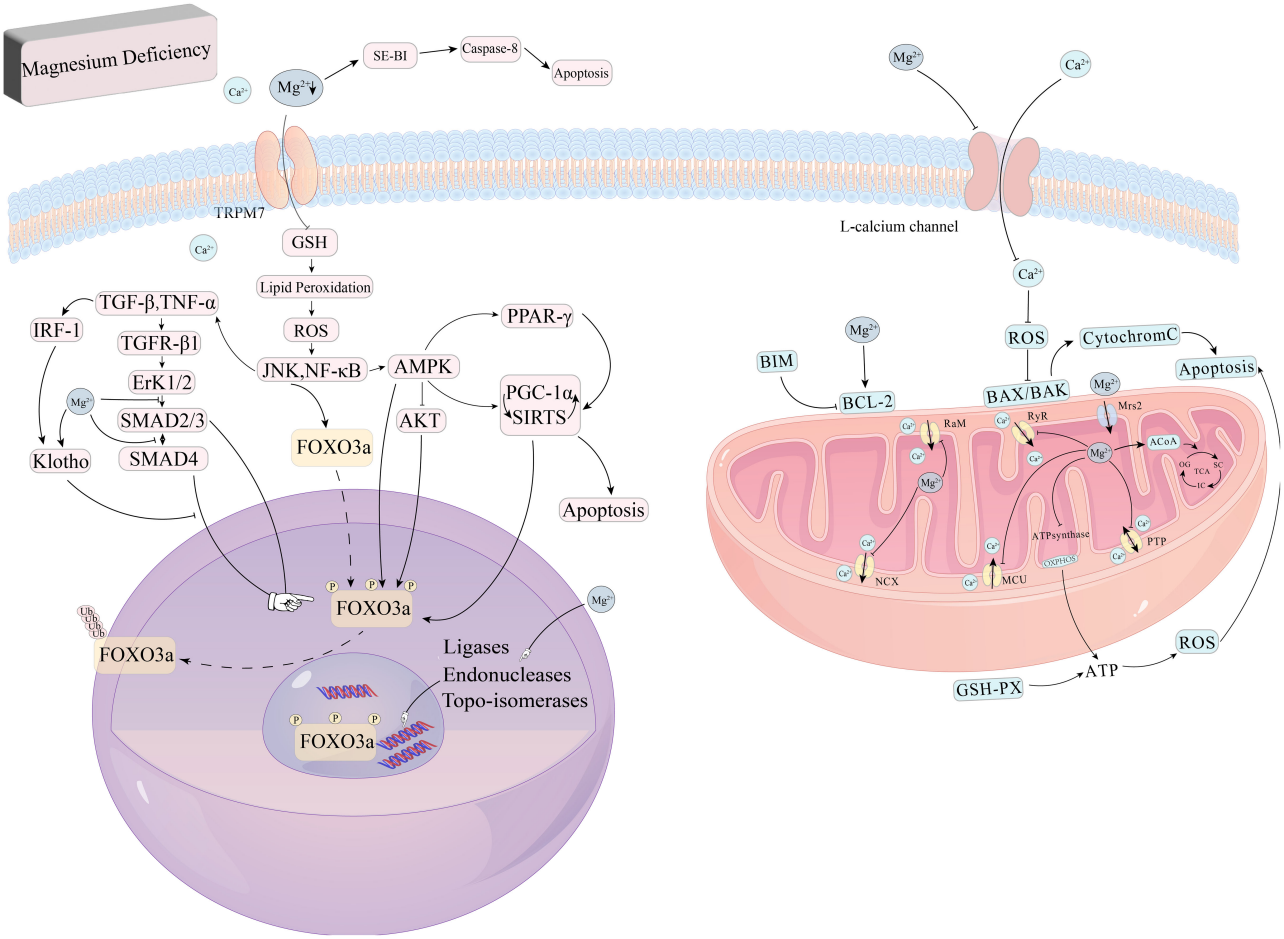
Role of magnesium in oxidative stress and apoptosis. Magnesium deficiency also triggers oxidative stress and disrupts the integrity of cell membranes, impairing DNA repair processes and contributing to apoptosis. In addition, Magnesium deficiency can also activate the caspase-8 signaling pathway to induce apoptosis and inhibit GSH activity, causing lipid peroxidation, excessive release of ROS, and directly or indirectly act on PGC-1α, SIRTs to induce apoptosis, in which SIRT1 and the AMPK signaling pathway can directly promote FoxO3a signaling pathway phosphorylation, and AMPK can inhibit the phosphorylation of FoxO3a by AKT to indirectly regulate FoxO3a. AKT phosphorylation of FoxO3a, which in turn indirectly regulates FoxO3a.

In summary, Magnesium regulates mitochondrial apoptosis, thereby protecting the kidney from damage during renal injury progression. Magnesium deficiency induces apoptosis and impairs cellular function (62, 63). Magnesium plays a central role in several key signaling pathways that inhibit renal apoptosis and fibrosis, as illustrated in **Figure 2**.

## 4 Mechanisms of magnesium regulating cell signaling pathway to improve DKD

Numerous signaling pathways influence DKD, with FoxO family proteins being of particular interest due to their significant role in DKD progression (64). Increasing evidence suggests that FoxO transcription factors have antioxidant activity, primarily reducing oxidative stress in DKD (64, 65). Recent research also indicates that the FoxO family plays a crucial role in DKD progression by inhibiting ferroptosis (66). Moreover, Magnesium can regulate the FoxO family through various signaling pathways, improving DKD outcomes.

### 4.1 The FoxO family and DKD

The FoxO family includes FoxO1 Forkhead in Rhabdomyosarcoma), FoxO3 (Forkhead in Rhabdomyosarcoma-like 1), FoxO4 (Acute lymphoid leukemia fused gene), and FoxO6 proteins. FoxO1 is predominantly expressed in adipose tissue, liver, skeletal muscle, pancreas, and brain. FoxO3 is primarily expressed in the liver, FoxO4 in muscle, and FoxO6 in the brain. These proteins are encoded by distinct genes but share a common DNA-binding domain. The FoxO domain consists of a forkhead region at the N-terminal, a transactivation region at the C-terminal, and nuclear localization/export domains that regulate its entry and exit from the nucleus (67). Post-translational modifications can activate or inhibit FoxO’s transcriptional activity by regulating its nuclear localization (68, 69). FoxO plays a crucial role in cellular processes such as proliferation, apoptosis, metabolism, oxidative stress, DNA repair, and cell cycle arrest.

DKD is characterized by structural and functional abnormalities of the kidneys in patients with diabetes. Structural abnormalities include benign renal hypertrophy, increased glomerular membrane thickness, interstitial fibrosis, and glomerulosclerosis, whereas functional changes encompass alterations in glomerular filtration rate, proteinuria, and albuminuria (70). Oxidative stress is a primary driver of DKD onset and progression. The study has shown that FoxO transcription factors are strongly correlated with DKD and are primarily involved in mitigating oxidative stress (64).

FoxO transcription factors are categorized into four main classes, with FoxO3a playing a crucial role in the progression of DKD. Given FoxO3a’s crucial role in maintaining ROS homeostasis, along with its widespread expression and endogenous characteristics, it provides new opportunities for studying antioxidant defense mechanisms.

#### 4.1.1 FoxO3a

**Figure 3:**
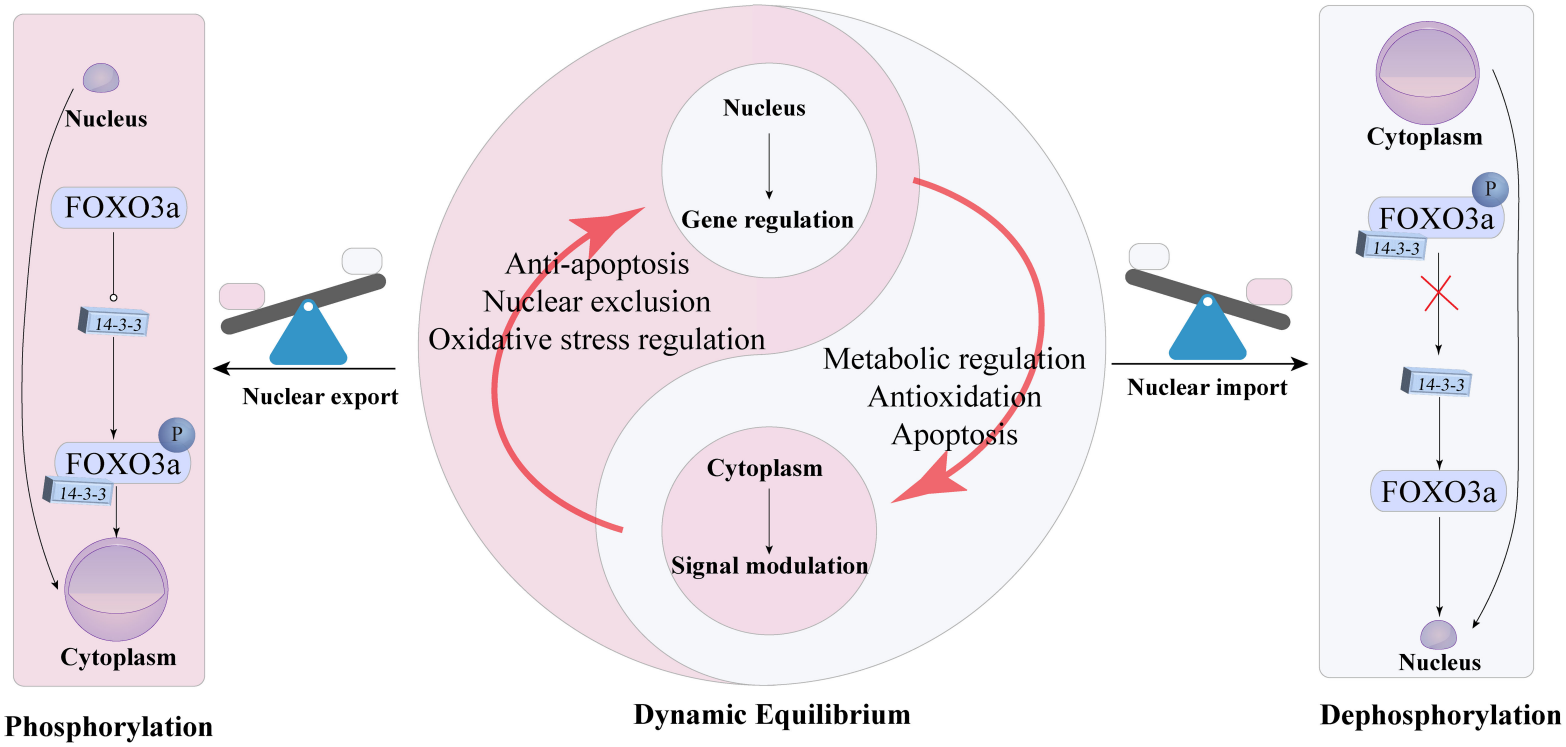
Dynamic equilibrium and functional switching mechanism of FoxO3a between the nucleus and cytoplasm of cells.

Functional studies have shown that phosphorylation and acetylation of FoxO3a often occur simultaneously and interact with each other (71). Non-phosphorylated FoxO3a remains in the nucleus, while phosphorylation via the PI3K/Akt pathway translocates it to the cytoplasm, inhibiting the transcriptional activation of its target genes (72). Additionally, p38β-mediated phosphorylation upregulates BCL-associated protein expression, leading to apoptosis (73). In mouse models of DKD, FoxO3a activation has been identified as a potential antioxidant mechanism for alleviating DKD, particularly through its translocation from the cytoplasm to the nucleus (74). FoxO3a can also be activated by SIRT1 and SIRT3, which deacetylate FoxO3a, enhancing its activity. Another animal study on DKD confirmed that FoxO3a transcription factors play a role in improving DKD (65). Under persistent hyperglycemia, excessive ROS accumulation upregulates TGF-β1, which activates the PI3K/Akt-FoxO3a pathway. This leads to FoxO3a phosphorylation and its binding with 14-3–3 proteins, resulting in cytoplasmic translocation and inhibition of its activity, exacerbating DKD progression (64), the relationship between FoxO3a and the nucleus is shown in **Figure 3**. Inhibition of TGF-β1 and PI3K preserves FoxO3a nuclear localization. Increased TGF-β1 expression in diabetes activates Akt, phosphorylating FoxO3a and causing its nuclear export, regulating FoxO3a activity through the TGF-β1-PI3K-Akt pathway. Thus, FoxO3a plays a crucial role in mitigating oxidative stress in DKD. Additionally, hypoglycemic drugs alleviate DKD by increasing FoxO3a and SIRT3 expression in hyperglycemic conditions (75). Resveratrol activates the AMPK-SIRT1-PPARα pathway, reducing FoxO3a phosphorylation and increasing its expression in the renal cortex, improving DKD symptoms (76).

Furthermore, FoxO3a plays a role in inducing cellular autophagy—a highly conserved process in eukaryotes responsible for recycling or degrading misfolded proteins and damaged organelles to maintain cellular homeostasis (77). Autophagy is a critical cellular process that regulates DKD progression. A recent study by Dusabimana et al. demonstrated that reduced expression of SIRT1 and FoxO3a inhibits autophagy gene induction and promotes DKD progression, whereas restoring FoxO3a and SIRT1 signaling pathways enhances autophagy, thereby reducing DKD-associated pathological changes (78).

### 4.2 Mechanisms of magnesium regulating the FOXO family to improve DKD

#### 4.2.1 Regulating the PI3K/Akt/FOXO3a pathway to alleviate oxidative stress

PI3K is a phosphoinositide kinase with three isoforms, of which PI3K I is the most common (79). PI3K is activated by growth factors, cytokines, hormones, tyrosine kinases, and G-protein-coupled receptors. In response to extracellular stimuli (e.g., insulin resistance), PI3K phosphorylates phosphatidylinositol 4,5-bisphosphate (PIP2), producing phosphatidylinositol-3,4,5-trisphosphate (PIP3), which leads to AKT translocation and activation, catalyzed by phosphoinositide-dependent kinase-1 (PDK1) (80). Activated PI3K also recruits signaling proteins, primarily AKT, to the intracellular membrane (81).

AKT, a serine/threonine kinase, is a central hub in numerous signaling pathways and a critical mediator of the PI3K signaling pathway. It exists in three isoforms: AKT I, ubiquitously expressed; AKT II, predominantly found in insulin-sensitive tissues; and AKT III, expressed in the testes and brain, regulating glucose and lipid metabolism (82, 83). AKT activation occurs via two key phosphorylation processes: PDK1 phosphorylates the kinase domain of AKT1, initiating activation, followed by phosphorylation of the C-terminal regulatory region by mTOR complex 2 (mTORC2) via a PI3K-dependent mechanism (84, 85). Activated AKT regulates cell survival, proliferation, migration, metabolism, and angiogenesis (86). In insulin-responsive tissues, AKT II promotes GLUT4 translocation to the plasma membrane (87). AKT activation further enhances PI3K/AKT signaling and regulates downstream molecules such as glycogen synthase kinase, increasing insulin sensitivity and protecting the vascular endothelium (88). In DKD, endothelial dysfunction and glomerular damage exacerbate proteinuria and promote DKD progression (89). Thus, alleviating oxidative stress and endothelial dysfunction via the PI3K/AKT pathway is crucial for improving DKD.

Magnesium regulates the PI3K/AKT signaling pathway in DKD. Recent studies have shown that Magnesium reduces vascular neointima formation after arterial injury by activating the antioxidant transcription factor Nrf2 through the PI3K/AKT pathway (90), thereby alleviating DKD progression. Additionally, Magnesium exerts anti-inflammatory effects in diabetes by enhancing PI3K/AKT activity (91). Magnesium also serves as a cofactor for the insulin receptor β-subunit, regulating tyrosine kinase activity in peripheral tissues. Insulin promotes glucose transport, glycogen synthesis, and protein synthesis through the PI3K/AKT signaling pathway (92). The insulin receptor comprises two α-subunits and two β-subunits. Upon insulin binding, tyrosine residues in the β-subunits are phosphorylated, activating complex intracellular signaling networks. Studies have shown that Magnesium enhances GLUT4 activation by promoting AKT activation and GLUT4 translocation, thereby regulating insulin signaling and glucose uptake in peripheral tissues (93). Magnesium also increases AKT gene expression in type 2 diabetic rats, thereby improving insulin resistance (94). Magnesium treatment improved AKT and PI3K phosphorylation levels in the brains of rats with renal failure (95). Impairment of the PI3K/AKT pathway in various tissues leads to insulin resistance and type 2 diabetes, which subsequently exacerbates PI3K/AKT pathway dysfunction, creating a vicious cycle (92). In summary, Magnesium mitigates DKD progression by upregulating the PI3K/AKT pathway, though the precise mechanism regulating downstream signaling requires further elucidation.

The PI3K/AKT pathway enhances cell proliferation and inhibits apoptosis, primarily by regulating its downstream transcription factor, FoxO3a (96). Under normal conditions, FoxO3a functions as a transcription factor in the nucleus, binding to DNA as a monomer. In the context of sustained hyperglycemia in DKD, ROS overproduction activates the TGF-β1-PI3K/AKT pathway, leading to FoxO3a phosphorylation, nuclear export, and inhibition of its normal transcriptional activity (65). FoxO3a regulates the transcription of several pro-apoptotic genes, including BIM, NOXA, TRAIL, PUMA, and FASL (97).

**Figure 4:**
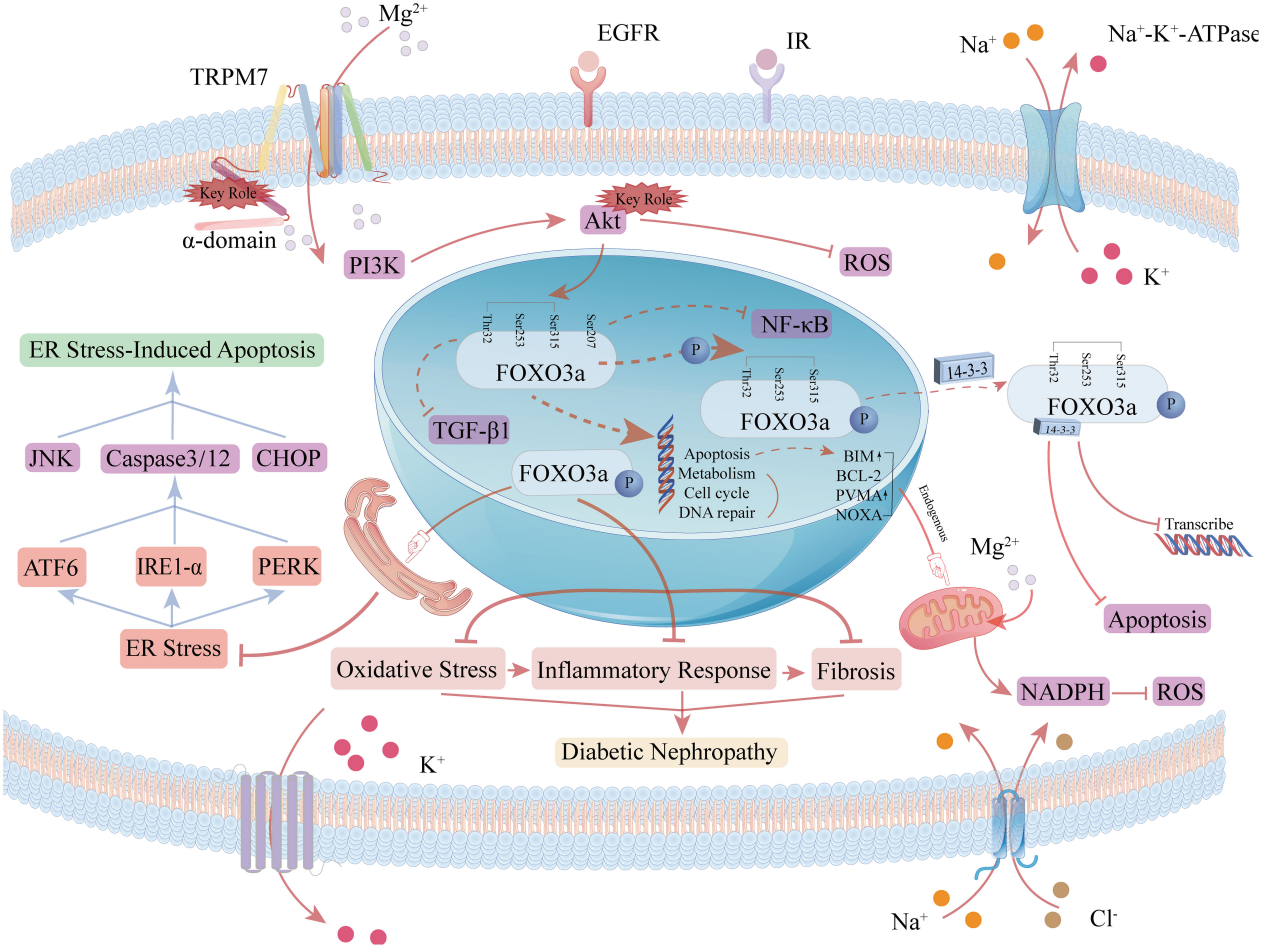
Role of the FOXO3a signaling pathway in oxidative stress and apoptosis. FoxO localization is induced by AMPK (AMP-activated protein kinase), p38 MAPK (p38 mitogen-activated protein kinase), Erk (extracellularly regulated kinase), JNK (c-Jun-N-terminal kinase), and MST1 (macrophage-stimulated gene 1), which increases FoxO transcriptional activity. When the PI3K signaling cascade is activated, the PI3K/Akt pathway in the FoxO3a signaling pathway phosphorylates FoxO3a at the sites of threonine residue 32 (Thr32), serine residue 253 (Ser253), and serine residue 315 (Ser315), and the phosphorylated FoxO3a binds to 14-3–3 proteins to form a complex, which translocates from the nucleus to the cytoplasm, reducing the ability of FoxO3a to bind to DNA and contributing to its accumulation in the cytoplasm as well as ubiquitination and degradation.

Therefore, elucidating the composition and interrelationships of the FoxO3a signaling pathway is crucial for understanding its redox nature and enabling precise therapeutic interventions. Investigating the regulation and mechanisms of FoxO3a under various pathophysiological conditions may offer new avenues for clinical DKD treatment. The pathways involving FoxO3a in oxidative stress are depicted in **Figure 4**. FoxO3a transcription factors are involved in various cellular processes, with their primary role in mitigating DKD progression being linked to antioxidant activity (64, 65). Further research is required to elucidate additional pathways through which FoxO3a transcription factors may contribute to inhibiting or slowing DKD progression.

#### 4.2.2 Regulating the AMPK/Fox3a/Nrf2 pathway to inhibit ferroptosis

AMPK is an enzyme widely expressed in the kidney, heart, and other tissues, serving as a sensor of cellular energy status. In the FoxO3a oxidative stress cascade, AMPK activates FoxO3a, enhancing its transcriptional activity and playing a crucial role in maintaining mitochondrial homeostasis. Upon activation, FoxO3a reduces hypoxia-inducible factor accumulation, thereby inhibiting ROS production (98). It has also been shown that AMPK/FoxO3a signaling activation, mediated by energy stress, inhibits ferroptosis through mitochondrial-dependent mechanisms (99). Additionally, AMPK directly inhibits fatty acid synthesis by suppressing acetyl-CoA carboxylase (ACC), thereby alleviating ferroptosis (99). Under persistent hyperglycemia in DKD, excessive Fe^2+^ accumulation increases ROS production, promoting ferroptosis (100). In DKD-related animal models, renal tubular injury has been identified as a key mechanism of DKD, with ferroptosis as its primary pathological process. Increased ferroptosis levels further exacerbate renal tubular injury, promoting DKD progression (42). A recent pharmacological study on fenofibrate demonstrated that restoring antioxidant capacity alleviates tubular damage and renal injury in DKD by upregulating Nrf2 expression, which in turn regulates GPX4, SLC7A11, ferritin heavy chain 1 (FTH-1), and transferrin receptor 1 (TFR-1) (39). Nrf2, a key protein in the pathological process of DKD, regulates numerous genes involved in iron storage and transport at the transcriptional level and acts as a downstream signaling molecule of the AMPK/FoxO3a pathway. Recent studies have shown that regulating the FoxO3a/Nrf2 axis promotes the expression of key anti-ferroptosis factors, such as System Xc-, GPX4, and GSH, thereby inhibiting ferroptosis in renal tubular epithelial cells (101, 102). Additionally, the AMPK signaling pathway enhances antioxidant effects by promoting Nrf2 nuclear accumulation (103). An Engel-Lenin-related study found that regulating the AMPK/Nrf2 pathway mitigates ferroptosis in renal tubules, thereby slowing DKD progression (104). Furthermore, the AMPK/FoxO3a pathway regulates mitochondria to alleviate ferroptosis, and Nrf2 is a critical regulator of mitochondrial function. Nrf2 knockdown impairs mitochondrial function, whereas Nrf2 activation enhances it, as demonstrated in animal experiments (105, 106). Nrf2 regulates MEF-2 to enhance mitochondrial fusion and fission processes and improve ferroptosis defense, thereby mitigating ferroptosis (107). Thus, the AMPK/FoxO3a/Nrf2 axis likely plays a crucial role in regulating ferroptosis in DKD. Magnesium, as a vital cofactor for GPX and GSH regulation, is essential for modulating the AMPK/FoxO3a/Nrf2 axis.

**Figure 5:**
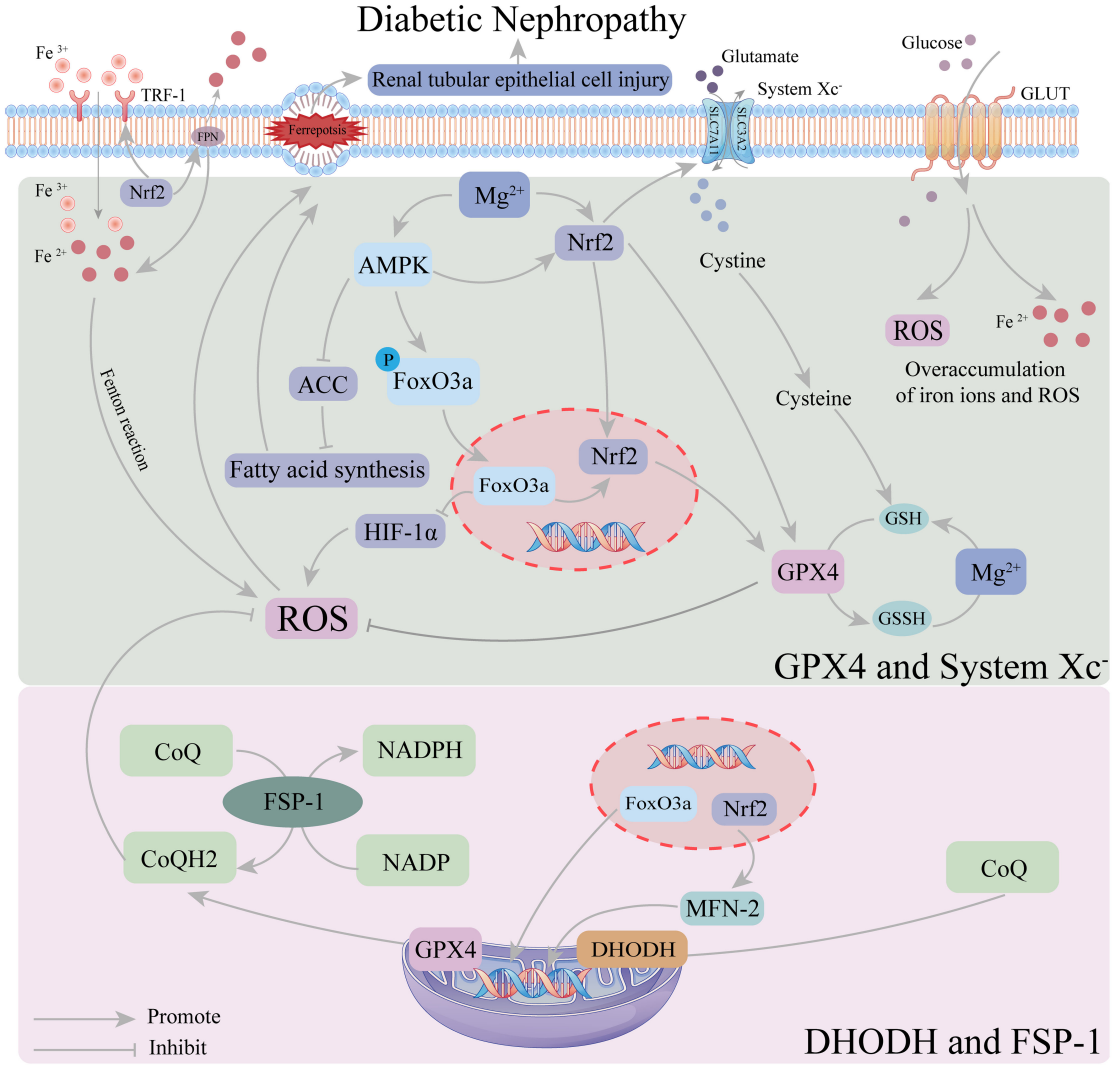
Mechanism of magnesium regulation of ferroptosis in the AMPK/FoxO3a/Nrf2 pathway. In the defense system of ferroptosis, GPX4 and system Xc-system occur mainly in the cytoplasm, while DHODH and FSP-1 occur mainly in the mitochondria. Magnesium can inhibit the HIF-1α-induced increase in ROS by up-regulating AMPK activity FoxO3a dephosphorylation. Magnesium upregulation of AMPK activity also promotes the intranuclear accumulation of Nrf2, which increases GPX4 activity and strengthens the ferroptosis defense system in the cytoplasm. Intranuclear FoxO3a and Nrf2 can also participate in the regulation of GPX4 and related coenzymes in mitochondria, thereby enhancing the ferroptosis defense system in mitochondri.

Recent studies have demonstrated that Magnesium directly upregulates Nrf2 expression, protecting cells from inflammatory damage and mitigating ferroptosis during DKD progression. Magnesium upregulates Nrf2 expression by inhibiting the PKC pathway, thereby activating the AMPK pathway and reducing lipid deposition (108, 109). It also enhances Nrf2 transcription (110) by inhibiting glycogen synthase kinase 3β (Gsk3β) (111, 112) and disrupting the interaction between Kelch-like ECH-associated protein 1 (Keap1) and Nrf2 (113), thereby enhancing Nrf2 activity. Additionally, Magnesium directly activates the AMPK pathway (114) and upregulates p-AMPK levels in a dose-dependent manner (115), indirectly increasing downstream Nrf2 expression. Thus, Magnesium regulates the AMPK/FoxO3a/Nrf2 pathway to mitigate ferroptosis in DKD, with the specific mechanism depicted in **Figure 5**.

## 5 Clinical treatment of DKD with magnesium

DKD is the leading cause of renal failure, and current clinical treatments for DKD focus on protecting the kidneys and reducing proteinuria. These drugs mainly include renin-angiotensin-aldosterone system (RAAS) blockers, angiotensin-converting enzyme inhibitors (ACE-Is), and angiotensin receptor blockers (ARBs), sodium-glucose transport protein 2 (SGLT2) inhibitors (116). However, the therapeutic efficacy of conventional drugs remains suboptimal, highlighting the urgent need for new therapeutic strategies. Trace elements are crucial for human metabolism and tissue function. In recent years, the biochemical functions of trace elements and their roles in disease management have been extensively studied (117). Previously, Magnesium was not widely used as a therapeutic agent for DKD; however, with advances in understanding the pathological mechanisms of DKD, Magnesium supplementation shows potential for alleviating or treating this condition.

### 5.1 Dietary magnesium supplementation

**Figure 6:**
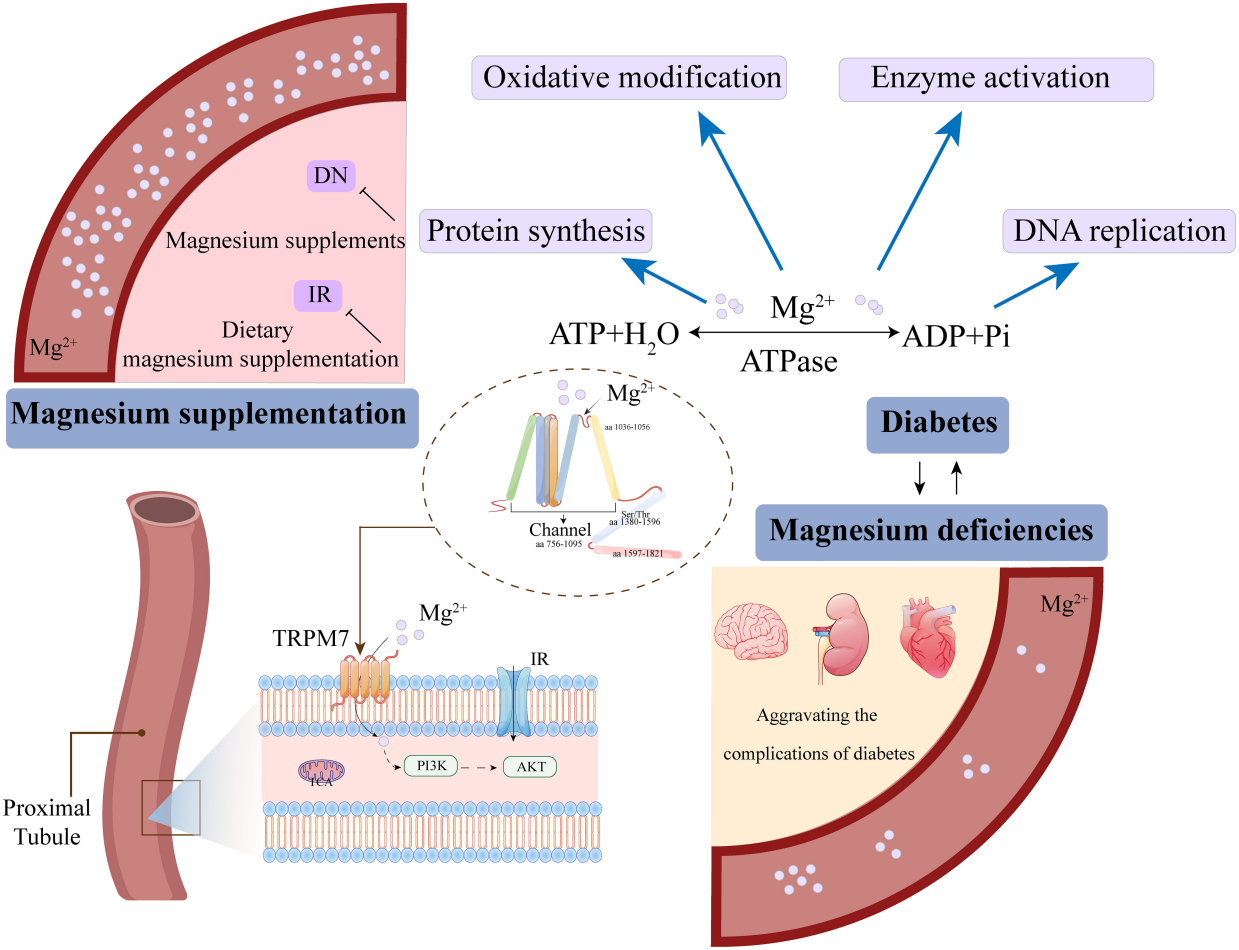
Magnesium ion deficiency and supplementation regulate homeostasis through cellular signaling.

Magnesium is of particular interest due to its critical role in glucose metabolism, insulin regulation, hypertension, inflammation, and cardiovascular health. A study on childhood obesity found that dietary Magnesium supplementation was inversely associated with insulin resistance (118). Additionally, lower dietary Magnesium intake was linked to increased insulin resistance and higher prevalence of both fasting pancreatic dysfunction and type 2 diabetes in adults (119, 120). The study demonstrated that Magnesium intake was inversely correlated with the risk of type 2 diabetes, with 17,130 participants followed for 28 years (121). Increasing dietary Magnesium intake has been shown to effectively prevent diabetes and improve DKD outcomes (122). Furthermore, increasing dietary Magnesium intake can reduce the risk of HDL cholesterol decline, decrease insulin resistance, and lower the risk of metabolic syndrome, thereby preventing DKD development (123). Adequate daily Magnesium intake helps restore optimal intracellular Magnesium levels, thereby enhancing insulin-mediated glucose uptake. A meta-analysis of numerous studies demonstrated that appropriate dietary Magnesium supplementation significantly improved insulin sensitivity and metabolic control in patients with type 2 diabetes and DKD (124). Conversely, an animal study found that restricted dietary Magnesium intake led to a chronic inflammatory response characterized by leukocyte and macrophage activation, increasing the risk of DKD (125). Thus, daily supplementation with moderate amounts of Magnesium may reduce the risk of DKD (126). For further elucidation, refer to **Figure 6**.

### 5.2 Magnesium supplements

In addition to dietary supplementation, Magnesium therapy has emerged as a key focus of diabetes research, demonstrating beneficial effects on diabetes mellitus and its complications in both humans and animal models (127, 128). A significant inverse association was observed between total magnesium intake and the risk of DN, exhibiting an L-shaped nonlinear dose–response relationship with a threshold of 345.00 mg/day (129). Magnesium supplementation can alleviate insulin resistance, decrease harmful metabolite levels, and improve blood glucose control in type 2 diabetes patients (130). Moreover, oral Magnesium supplementation significantly improves abnormal insulin secretion, stimulates pancreatic cells, promotes insulin release, enhances insulin sensitivity in peripheral tissues of patients with type 2 diabetes and hypomagnesemia (131), and regulates lipid levels, metabolism, and inflammation (132).

The study demonstrated that oral Magnesium citrate supplementation significantly alleviates microalbuminuria in patients with DKD, where microalbuminuria serves as a key indicator for DKD progression (133). Furthermore, Magnesium citrate supplementation demonstrated positive effects on serum osteocalcin, lipid profiles, and patient quality of life, without significant side effects (133, 134). A study by Morakinyo et al. demonstrated that Magnesium supplementation regulated GLUT4 translocation and improved metabolic status in rats (135). Magnesium supplementation also alleviated renal injury in a mouse model of DKD, delayed diabetes progression in a type 2 diabetes mouse model, enhanced mitochondrial function, and reduced oxidative stress (136). Moreover, oral Magnesium phosphate supplementation protects renal function, modulates the intestinal microbiota, restores metabolite dysfunction (e.g., short-chain fatty acids, amino acids), and inhibits binding of cresyl sulfate precursors, thereby reducing plasma uremic toxin levels in DKD mice and improving DKD (137). Therefore, more results from clinical studies are needed to recommend Magnesium as a treatment option for DKD.

## 6 Conclusion

The pathophysiology of DKD is complex and multifactorial, with oxidative stress, ferroptosis, and chronic inflammation playing key roles in its progression. Magnesium, as an essential cofactor for antioxidant enzymes, is closely linked to DKD, and Magnesium supplementation represents a promising therapeutic approach. Magnesium ions exhibit multiple renoprotective mechanisms to ameliorate DKD, including alleviating oxidative stress and inflammation, protecting endothelial cells, and maintaining vascular integrity through the PI3K/AKT/FoxO3a pathway. Additionally, Magnesium alleviates ferroptosis in renal tubular epithelium and podocytes, maintaining tubular integrity via the AMPK/Nrf2/FoxO3a pathway. Clinical studies have demonstrated that Magnesium supplementation, both dietary and pharmacological, exerts positive effects in patients with DKD. However, further studies are needed to fully evaluate Magnesium ‘s potential as a clinical treatment for DKD and to elucidate the underlying mechanisms of Magnesium action.

This review explores the relationship between Magnesium and DKD, emphasizing the effects of Magnesium deficiency, as well as the impact of dietary Magnesium supplementation and Magnesium supplements on DKD. The mechanisms through which Magnesium delays DKD—by alleviating oxidative stress, inhibiting ferroptosis, and improving mitochondrial function—are detailed. Detailed discussion is provided on Magnesium regulates DKD via the FoxO family, laying the groundwork for the use of Magnesium in the treatment and management of DKD.
